# Nutritional Status, Selected Nutrients Intake and Their Relationship with the Concentration of Ghrelin and Adiponectin in Patients with Diabetic Nephropathy

**DOI:** 10.3390/nu13124416

**Published:** 2021-12-10

**Authors:** Iwona Boniecka, Anna Jeznach-Steinhagen, Weronika Michalska, Aleksandra Rymarz, Dorota Szostak-Węgierek, Stanisław Niemczyk

**Affiliations:** 1 Department of Clinical Dietetics, Faculty of Health Sciences, Medical University of Warsaw, E Ciołka Str. 27, 01-445 Warsaw, Poland; asteinhagen@wum.edu.pl (A.J.-S.); dietetyk.michalska@gmail.com (W.M.); dorota.szostak-wegierek@wum.edu.pl (D.S.-W.); 2 Department of Internal Medicine, Nephrology and Dialysis, Military Institute of Medicine, Szaserów Str. 128, 04-141 Warsaw, Poland; arymarz@wim.mil.pl (A.R.); sniemczyk@wim.mil.pl (S.N.)

**Keywords:** chronic kidney disease, diabetic nephropathy, diet, ghrelin, adiponectin

## Abstract

Background: Overnutrition is one of the risk factors of chronic kidney disease (CKD). The factors related to both obesity and CKD are adiponectin and ghrelin. The aim of the study was to assess if there is a link of nutritional status and selected nutrients intake with adiponectin and ghrelin in patients with diabetic nephropathy (DN). Methods: The study involved 55 patients diagnosed with DN in the pre-dialysis period (two groups: GFR < 30 and >30 mL/min/1.73 m^2^). In all participants standard blood tests, total ghrelin and total adiponectin plasma concentrations and anthropometric measurements (BMI, WHR- waist–hip ratio, body composition analysis) were performed. The evaluation of energy and nutrient intakes was made using the three-day food record method. Results: Excessive body weight was found in 92.80% patients. The average daily energy intake was 1979.67 kcal/day (14.45% protein energy, 28.86% fat, and carbohydrates 56.89%). In the group with eGFR < 30 mL/min/1.73 m^2^ the analysis showed a negative relationship between ghrelin and WHR value, and the creatine and albumin concentrations. There was a positive correlation between ghrelin concentration and the consumption of carbohydrates and sucrose. In the group of patients with eGFR > 30 mL/min/1.73 m^2^, a positive correlation was found between the concentration of ghrelin and the consumption of vegetable protein, carbohydrates, and glucose. Conclusions: The study confirms the high prevalence of obesity in patients with DN-Excessive supply of protein was found in the patients’ diets, which may contribute to the deterioration of the course of the disease and its prognosis. In patients with eGFR < 30 there was a negative correlation between ghrelin concentration and nutritional status, and in patents with eGFR > 30 between ghrelin concentration and some nutrients intake.

## 1. Introduction

Globally in 2017, there were 697.5 million cases of CKD (chronic kidney disease). Thus, the prevalence of CKD is higher than that of diabetes, osteoarthritis, chronic obstructive pulmonary disease (COPD), asthma, or depressive disorders. The prevalence of CKD was estimated as 9.1% in the world’s population, with CKD Stages 1–2 accounting for 5.0%, Stage 3 for 3.9%, Stage 4 for 0.16%, Stage 5 for 0.07%, dialysis patients for 0.041%, and those after kidney transplantation for 0.011%. Since 1990 the global all-age prevalence of CKD increased by 29.3%. In 2017, 1.2 million people died from CKD. It is predicted that in 2040 this number may increase to 4.0 million [1].

Kidney disease has a major effect on global health, both as a direct cause of global morbidity and mortality and as an important risk factor for cardiovascular disease [1]. In recent decades, it was shown that CKD is not only a consequence of various diseases but also a predictor of the progression of certain cardiovascular diseases and mortality, the combination of which is constantly growing [2]. The leading risk factors for CKD are hypertension, diabetes and obesity, which are all modifiable [2]. It is estimated that >24% of CKD cases in industrialized countries can be attributed to nutritional factors, the most important of which are calorie dense diet, high in animal protein and sodium but low fruit and vegetable consumption [3]. In recent years, there is a growing body of evidence of the role of fructose (derived from fruits, honey, high-fructose corn syrup) in the pathogenesis and course of chronic kidney disease, because excessive fructose consumption has been shown to promote hypertension and insulin resistance [4,5,6,7,8,9,10,11].

The factors related to both metabolic disorders, including obesity and chronic kidney disease, are adiponectin and ghrelin. Adiponectin is a cytokine produced by adipose tissue [12,13,14,15,16,17,18,19,20,21]. It is cleared rapidly from the circulation primarily by the liver, involving in part the biliary route, and secondarily by the kidneys [12,13,14,16].

Adiponectin has been shown to have anti-inflammatory, antifibrotic and antioxidant effects on kidneys through AMP-activated protein kinase (AMPK-activated protein kinase) activation. It has been found that adiponectin receptor ADIPOR1 and catalytic AMPK subunits are expressed in the cells constituting the glomerulus [12,14,17,20,21,22]. Renal tubular cells can express and secrete adiponectin, which may contribute to some extent to the total amount of urinary adiponectin [12,14,15,16]. Serum adiponectin levels are significantly lower in type 2 diabetes (T2D), obesity, metabolic syndrome, and atherosclerosis. Despite high prevalence of metabolic disorders (e.g., insulin resistance) patients with renal disease have paradoxically higher serum adiponectin levels than subjects with normal kidney function [12,13,23]. What is more, elevated adiponectin concentration in established chronic kidney disease positively predict progression of disease [13,16,18,19,20,21,23], what can be associated with the fact that kidneys express and secrete adiponectin which is increased upon inflammatory stimulus [15,19,20,21].

Ghrelin, a peptide hormone released mainly by the X/A-like cells of the gastric mucosa, plays an important role in hunger regulation [2,22,24,25,26]. Increase in ghrelin level stimulates the appetite leading to obesity and kidney damage, i.e., ghrelin is metabolized and excreted by the kidneys and plays an important role in the pathogenesis of protein balance changes, inflammation and cardiovascular complications in CKD [2,22,23,24]. A direct relationship was found between the increased fasting ghrelin levels (1,4 times) in patients with stage 2 CKD and the background obesity [2]. On the other hand, recent studies have suggested that in CKD patients elevated total ghrelin levels is observed, what is due to increased concentration of anorexic form—DAG (des-acyl ghrelin) levels (one of the three circulating ghrelin forms) [2,22,24,25,27].

It should be emphasized that CKD is preventable and treatable to a large extent [1]. An important role in these processes is played by an appropriate diet, which supports treatment and prevents both obesity and malnutrition [3,27,28,29,30,31,32,33,34,35,36,37,38,39,40,41,42,43].

The aim of the study was to assess if there is a link of nutritional status and selected nutrients intake with the concentration of adiponectin and ghrelin levels in patients with diabetic nephropathy depending on the renal function.

## 2. Materials and Methods

The research was conducted at the Department of Internal Diseases, Nephrology and Dialysis of the Military Institute of Medicine and at the Department of Clinical Dietetics of the Medical University of Warsaw. The study involved 55 patients (including 32 men and 23 women) with a mean age of 70.7 ± 12.1 years diagnosed with chronic kidney disease in the pre-dialysis period, Stages 2 to 4. Patients were divided into two groups depending on the degree of renal efficiency, GFR < 30 and >30 mL/min/1.73 m^2^. To assess the degree of renal function, the MDRD formula proposed by the Modification of Diet in Renal Disease Study Group was used [44], which, in accordance with the joint recommendations of the National Consultant in Nephrology team and the General Board of the Polish Nephrology Society, for general use by adults [45]. Twenty-four patients were treated with insulin and 31 with hypoglycemic drugs.

All patients underwent blood tests and anthropometric measurements that included body composition, and also dietary assessment that comprised intake of energy and selected nutrients.

### 2.1. Anthropometric Measurements

Body weight (BW) was measured with high-quality electronic calibrated scale and height with a wall-mounted stadiometer (to the nearest 0.5 cm). Body mass index (BMI) was calculated as weight in kilograms divided by the square of height in meters [46].

In addition, waist, and hip circumference (WC and HC) were measured. Based on these results waist–hip ratio (WHR) and waist-to-height ratio (WHtR) were calculated. According to WHO recommendations [47] waist circumference (WC) was measured at the midpoint between the lower margin of the least palpable rib and the top of the iliac crest, using a stretch-resistant tape. Hip circumference was measured around the widest part of the buttocks. The waist–hip ratio (WHR) was calculated by dividing the waist circumference by the hip circumference. Abdominal obesity is defined as waist–hip ratio above 0.90 for males and above 0.85 for women [47]. The cut-off points of the WC, for the European population, are 94 cm and 80 cm respectively [47,48] The waist-to-height ratio (WHtR) was calculated by dividing WC by height. WHtR ≥ 0.5 is considered to be the early indicator of health risk [49,50].

Body composition analysis was performed based on bioimpedance spectroscopy (BIS) using a Body Composition Monitor (BCM Fresenius Medical Care). Measurements were conducted after a 5-min rest in a supine position. Pairs of electrodes were placed on one hand and on one foot. Among BIS measurements there were lean tissue mass (LTM), fat mass (FM) values. The average content of adipose tissue and lean tissue in the body for people aged ≥ 70 is 20–22% and 75–80% for men, and 28–36% and 70–75% for women, respectively [51].

### 2.2. Blood Tests

Blood tests included measurement of the concentration of creatinine, urea, total protein, albumin, prealbumin, lymphocytes, phosphorus, CRP, total adiponectin and total ghrelin. Blood samples were collected after an overnight fast (8–14 h after the last meal). Phosphorus levels were measured using the appropriate chemical analyzer (Cobas c501, Roche Diagnostic, Rotkreuz, Switzerland). Serum creatinine concentrations (SCr) were analyzed using Jaffe method (Gen.2, Roche Diagnostics GmbH, Rotkreuz, Switzerland), serum albumin levels (SA) using a BCP Albumin Assay Kit (Roche Diagnostics GmbH, Rotkreuz, Switzerland), and concentrations of high-sensitivity C-reactive protein were determined by a nephelometry assay (BN™ II System Siemens). All the above-mentioned blood tests were performed at the local Department of Laboratory Diagnostics.

Total ghrelin and total adiponectin plasma concentrations were measured at the Department of Experimental and Clinical Physiology, Laboratory of Centre for Preclinical Research, Medical University of Warsaw, by using enzyme-linked immunoassays (ELISA): ELISA Kit for Ghrelin (GHRL) CEA991Hu; Cloud-Clone Corp. (detection range: 123.5–10,000 pg/mL; sensitivity: <49.5 pg/mL; reference ranges: normal weight/control subjects: 520–700 pg/mL; obese subjects prior to diet: 340–450 pg/mL), and Adiponectin ELISA, E09; Mediagnost (assay range 0.27–31,000 ng/mL; sensitivity < 0.27 ng/mL; reference ranges for females: 1.121 × 10^4^–2.410 × 10^4^ ng/mL, for male: 3000–2.11 × 10^4^ ng/mL) and according to the manufacturer’s instructions. Mediagnost E09 Adiponectin ELISA detects all forms of Adiponectin present in human serum: the trimer at 65 kDa, the hexamer at 150 kDa and the high molecular weight forms of >280 kDa. The Mediagnost E09 Adiponectin ELISA therefore measures total Adiponectin.

### 2.3. Dietary Assessment

The evaluation of energy and nutrient intakes was made using the three-day food record method. The participants were asked to write down amounts of all foods and drinks they consumed for three days (including one weekend day). During a visit to a dietitian, the sizes of individual meals were precisely evaluated. To assess the nutritional value of patients’ diets the Dieta 5.0 software was used (launched by National Food and Nutrition Institute, Warsaw, Poland). The calculated nutritional data included: energy (kcal), total protein (grams) including animal and vegetable protein (g), carbohydrates (grams) including sucrose, glucose and fructose (grams), fiber (grams), fats (grams), potassium (grams), and phosphorus (grams) intake.

### 2.4. Statistical Analysis

The analysis of the results included the analysis of the relationship between the variables and the verification of statistical significance of intergroup differences. Pearson’s r correlation coefficients were used in the analysis of the relationship between the variables. The verification of the statistical significance of intergroup differences was based on the values of the Student’s *t*-test for independent samples.

## 3. Results

### 3.1. Anthropometric and Blood Parameters, Nutrients Intake

The study was conducted among 55 patients including 32 men and 23 women. The mean value of BMI was 30.68 ± 6.40 kg/m^2^. BMI value indicating excessive body weight (≥25 kg/m^2^) was found in 92.80% of the participants. Overweight and obesity concerned a similar percentage of subjects (47.3% and 45.5%, respectively). Abdominal obesity based on waist circumference and WHR was shown in 89.09% of participants (87.50% of men and 95.65% of women) and in 92.73% (96.88% of men and 86.96% of women) respectively. Based on the WHtR, a high metabolic risk was found in 8.2% of patients. The average content of adipose tissue in the body of the subjects was 41.03 ± 9.08%. Detailed data on the results of anthropometric measurements of patients is presented in Table 1.

The mean value of the eGFR MDRD was 27.99 ± 10.50 mL/min/1.73 m^2^, creatinine 2.44 ± 0.93 mg/dL, and urea 92.64 ± 32.04 mg/dL. The ghrelin concentration was 171.45 ± 44.44 pg mL and the adiponectin concentration was 2709.74 ± 2433.66 ng/mL (median 1818.34 ng/mL). The detailed results are presented in Table 2.

In the group with eGFR > 30, the mean concentration of creatinine and urea was lower than in the eGFR < 30 group (1.77 mg/dL vs. 2.76 mg/dL and 66.75 mg/dl vs. 105.29, respectively, *p* < 0.0001) and the concentration of albumin was higher, at borderline of significance (4.26 g/dL and 4.11 g/dL, respectively; *p* = 0.07). There were no statistically significant differences in other blood parameters between two groups.

Based on the analysis of three-day dietary records it was shown that the average daily energy intake was 1979.67 kcal/day. The percentage of the total calorie intake from proteins was 14.25%, from fats 28.86%, and from carbohydrates 56.89%. The consumption of total, vegetable and animal protein was 0.84 g/kg body weight, 0.30 g/kg body weight, and 0.54 g/kg body weight, respectively. Other information about selected nutrients intake is presented in Table 3.

The average daily nutrients intake was similar in both compared groups (eGFR < 30 and >30).

### 3.2. Correlations of Ghrelin and Adiponectin with Other Parameters Tested

The analysis of the correlation between selected anthropometric parameters and ghrelin concentration in two groups of patients of different stage of the disease showed that among those with GFR value < 30 mL/min/1.73 m^2^ there was a significant negative relationship between the ghrelin concentration and WHR value (Table 4).

The assessment of the relationship between the concentration of ghrelin and adiponectin from one side and other blood parameters on the other, in two groups of patients of different stage of the disease (with eGFR < 30 and >30 mL/min/1.73 m^2^) showed a negative correlation (*p* < 0.05) between the concentration of ghrelin and the concentration of creatinine (−0.353) and albumin (−0.378) in the blood serum of patients with eGFR < 30 mL/min/1.73 m^2^. In both studied groups, no statistically significant relationships were found between the concentration of adiponectin and other blood parameters.

The relationship between ghrelin and adiponectin and the energy and nutrients intakes was also assessed. In the whole group a statistically significant positive correlation was found between ghrelin concentration and the consumption of carbohydrates and sucrose. Additionally, in the group of patients with eGFR > 30 mL/min/1.73 m^2^, a positive correlation was found between the concentration of ghrelin and the consumption of vegetable protein (*p* = 0.01), carbohydrates (*p* = 0.05), glucose (*p* = 0.05), fiber (*p* = 0.05) and potassium (*p* = 0.05) (Table 5).

## 4. Discussion

This study confirms a high prevalence of excess body weight (especially abdominal obesity) in patients with diabetic nephropathy may complicate the treatment of chronic kidney disease, accelerate the progress and aggravate the course of CKD [1,2,3,24,27,28,30,31,33,34,35,36,37,38,39,40,41,42,43].

It should be emphasized that the average waist circumference and WHR indicated abdominal obesity [47,48,49], and the average WHtR confirmed a high metabolic risk [47,48,49,50]. Although there was a high prevalence of excessive body weight no relationship was found between (ghrelin) concentration, body weight and BMI. However, in patients with GRF < 30 mL/min/1.73 m^2^, a negative relationship between ghrelin concentration and WHR was demonstrated. This result seems surprising considering that ghrelin is an appetite-enhancing hormone. However, the study did not distinguish between the forms of ghrelin. It should also be emphasized that results of other studies differ in terms of the levels of acyl ghrelin (AG), with some studies suggesting higher levels in CKD patients whereas others show no difference [26,52]. According to Canpolant et al. [52] in patients with CKD, the appetite suppressant des-acyl ghrelin was the predominant form because it was found that plasma levels of total and des-acyl ghrelin, but not acyl ghrelin, increase in patients with CKD. Total ghrelin predominantly represents the des-acyl form of ghrelin which has anorexigenic effect and suppresses food intake what contributes to poor nutritional status [52].

It was worrying that there was a large proportion of fat tissue in the body composition of the subjects, which, together with a low proportion of lean body mass (mainly muscle tissue), may indicate sarcopenic obesity, common in patients with CKD [27,34]. It should be emphasized that the fat tissue increases, and lean tissue decreases, e.g., because physical activity declines with age and disease progression [27,29,34]. Abnormalities in body composition contribute to metabolic disorders-intensification of catabolism in the muscles and an increase in anabolism in adipose tissue. Moreover, the developing metabolic acidosis intensifies the degradation of muscles, as the natural mechanism protecting against acidosis is the release of glutamine from the muscles [27,32,53]. The progressive disproportion in body composition in favor of adipose tissue aggravates the often-existing insulin resistance. The loss of muscle mass is one of the indicators of malnutrition that occurs in approximately 20% of patients with chronic kidney disease [27,53]. Obesity, especially sarcopenic, due to the deficiency of muscle tissue, is considered one of the forms of malnutrition [26,32].

In the group of patients with eGFR < 30 mL/min/1.73 m^2^ we found a negative correlation between the concentration of ghrelin on one side and albumin and creatinine on the other. However, in our study we did not analyze the forms of ghrelin. The relation to albumin concentration can be explained by the already mentioned possible higher concentration of des-acyl ghrelin and low plasma levels of acyl ghrelin what may contribute to malnutrition [26,52] and this is associated with a poorer nutritional status in advanced stages of CKD [23]. The acylation is essential for its orexigenic and adipogenic effects, and ghrelin activation is suggested to be impaired in patients with CKD [25]. These corresponds to the results of our study. Lower albumin concentration was demonstrated in the group with eGRF < 30. Obviously, we cannot exclude proteinuria as a confounding factor.

The negative link of ghrelin to the concentration of creatinine is very surprising. Other authors showed that ghrelin levels positively correlated with serum creatinine and negatively with eGFR [23,54]. Increased total ghrelin levels in CKD are primarily due to its reduced renal clearance or degradation in CKD but without equal increase in AG level (orexigenic form) [22,24,26,52]. However, the study of Sekar et al. was conducted in hemodialysis patients. The mean BMI in the group was normal (22 kg/m^2^), and the energy and protein intake was 1369 kcal and 35 g/day, respectively, i.e., it was half that in our group of patients [54]. In another study, the plasma des-acyl ghrelin, but not total ghrelin, was significantly correlated with the serum creatinine level and was increased 2.8-fold in patients with end-stage renal disease compared with those in patients with normal renal function [22]. It is, thus, difficult to explain the negative correlation between ghrelin and creatinine in our patients with eGFR < 30. We suspect that in some of our patients the AG/DAG ratio was not disturbed, and hence the anorexigenic effect of the latter was not so pronounced, which made the impact on the disease progression smaller. It should also be considered that the group of diabetic patients with eGFR < 30 mL/min/1.73 m^2^ is a selected group of patients with advanced kidney failure which can be associated with metabolic disorders. On the other hand, diabetes also induces neurological and metabolic disturbances. Therefore, we can suppose that this relationship can be different than in other studies. We also cannot rule out that this result was influenced by the medications taken by the patients. This finding suggests the need for further studies in a larger group of patients, including analyzes of both forms of ghrelin

There was no correlation between the concentration of adiponectin and the results of anthropometric measurements and blood tests. In the El-Khashab and Behiry [23] study adiponectin was inversely correlated only with BMI in CKD patients. Song et al. demonstrated that an increased serum adiponectin level was significantly associated with higher serum creatinine level, lower BMI, reduced eGFR, and lower serum albumin level [21]. In other studies, it was found a negative correlation between adiponectin levels and visceral adipose tissue storage. Therefore, it is possible that visceral fat loss can increase adiponectin levels in overweight subjects [14]. It should be emphasized that though hypoadiponectinemia is an important risk factor for metabolic disorders [13,21], adiponectin levels are higher in CKD patients and have been associated with CKD progression [12,16,19,20,21]. Song et al. speculate that serum adiponectin is a biomarker of renal dysfunction rather than a true risk factor, intimately involved in CKD progression [21]. For this reason, adiponectin might be considered as a marker of kidney injury and risk of disease progression [12,16,21].

The significant prevalence of obesity in the study group did not reflect current excessive energy intake (1980 kcal/day). However, energy intake was higher than in other studies conducted among patients with CKD where it was in the range 1334 [43]–1394 [31] kcal/day. Therrien et al. [40] emphasize that most of the research on dietary intake is based on self-recording of food consumption by patients, and this method is associated with a high risk of underestimate. However, although the study group was dominated by patients with excess body weight, and the results of blood tests did not indicate malnutrition, it does not exclude that if the current diet was maintained or the study was carried out in a larger group, the results would be different. It has been shown that the higher the ghrelin concentration was, the higher the intake carbohydrates and sucrose was. In the group of patients with eGRF > 30 mL/min/1.73 m^2^ also the higher was the intake of vegetable protein, fiber, glucose, and potassium. This result suggests an orexigenic effect of ghrelin in the group of patients with a lower disease progression what corresponding with better nutritional status (higher albumin concentration) in this group. Both total carbohydrates as well as sucrose and glucose are a good source of easily and quickly available energy, especially in the case of hunger. However, frequent consumption of these products may contribute to excess body weight, and with insufficient consumption of other nutrients, especially protein, for malnutrition. Sucrose accounted for 9.9% of the energy value of the diet, total carbohydrates about 60%. In other studies, percentage of the total calorie intake from carbohydrates was both lower [43] and higher [31] than in ours. At the Gebretsadik at al. [31] study about 70% of the respondents had their total carbohydrates intake above recommended level. The sucrose consumption was almost the same (9.3% of total energy) [31]. Although the guidelines for CKD patients do not specify exactly what the supply of simple carbohydrates should be, it is usually assumed that the supply of total carbohydrates should be in the range of 45–60% of energy [28,35,55]. According to WHO recommendations the intake of carbohydrates from sugars (free sugars) should be reduced to less than 10% and preferably below 5% of total energy intake [56] and higher glycemic index foods should be substituted with low glycemic index, high fiber content foods such as multigrain cereal, and vegetables [33,35,36,37]. Low- glycemic index foods may improve overall blood glucose control [36] and this way reduce the risk of type 2 diabetes [57]. Currently, there are no specific recommendations for levels of dietary fiber intake for adults with CKD, but recommendations for the general population are likely safe and probably beneficial as long as serum potassium and phosphate levels are within the norms. Constipation in patients with CKD can lead to higher retention of uremic toxins, hyperkalemia, disturbances in intestinal microflora. Urea may alter directly the gut microbiome, disrupt the gut barrier function by reducing the presence of specific proteins in the tight junctions of the gut), increasing translocation of gut bacteria to the systemic circulation, and heightening inflammation, which may aggravate the course of the disease. Therefore, high-fiber diet may be protective in CKD by promoting the growth of commensal bacteria such as Bifidobacterium, an endosymbiotic colonizer of the gut that strengthens the gastrointestinal permeability barrier. It can increase the number of bowel movements therefore is associated with less uremic toxin production, exposure, and absorption and helps promote urea and potassium excretion [3,33]. That is why fiber intake may be important for patients with advanced CKD when urea excretion is severely impaired due to low GFR [3].

It should be emphasized that products rich in complex carbohydrates are an important source of vegetable protein. An increase in the percentage of calories from complex carbohydrate food may result in improvement in the outcomes of CKD and its accompanying comorbid conditions [38].

Fructose intake in the course of kidney disease is also important. In the past, fructose intake was provided by fruits and honey and its daily intake was 16–20 g. However, in the last 30 years, daily fructose intake has increased up to 85–100 g due to the addition of sucrose, high fructose corn syrup (HFCS), honey, to carbonated beverages, fruit juices, canned fruits, and dairy products [7]. One of the causes of obesity and its complications (including type 2 diabetes, hypertension and CKD) is the excessive consumption of sugar added to products, particularly those containing fructose. Sucrose is also a substantial source of fructose [7]. In our study, fructose consumption was 15 g/day, which corresponded to 2.8% of the energy value of the diet. In the study by Arslan and Sanlier [7], diabetic patients consumed about 13 g of fructose, which corresponded to about 7% of the energy value of the diet. According to Polish Guidelines [56] daily fructose intake should not exceed 50 g. Fructose use as a replacement for sugar is not recommended. It was reported that moderate intake of fructose (0–50 g/day) has potential benefits for glycemic control while high and very high intake of fructose may lead to risks of dysglycemia, insulin resistance and dyslipidemia [10]. What is more, excessive fructose consumption may be related to CKD due to its effect on hypertension and hyperuricemia [4,5,6,8,9]. Fructose may be filtered into the urine where it is taken up in the S3 segment of the proximal tubule, leading to local intracellular generation of uric acid with oxidative stress and local inflammation [8].

Taking into account the adverse effect of excessive protein intake on the kidneys (e.g., vasodilation of afferent renal arterioles, glomerular hypertension and hyperfiltration, increased production of uremic toxins, intensification of metabolic acidosis [3,30,33] and increased albuminuria [29], it should be noted that in the study group the protein supply was slightly increased (0.84 g/kg bw/day). This result is consistent with that obtained in a study conducted in a group of predialysis female patients [43]. Gebretsadik et al. [31] showed that the average dietary protein intake (DPI) was even higher (0.95 g/kg/day) then the recommended levels. In the updated KDOQI (Kidney Disease Outcomes Quality Initiative) Clinical Practice Guideline for Nutrition in CKD, it is highlighted that the low-protein diet (0.55–0.6 or 0.28–0.43 g/kg/day with keto-acid analogs) should be prescribed for nondiabetic and metabolically stable patients with Stages 3 and 4 CKD. This should be performed under close clinical supervision, preferentially by a dietician to reduce any risk that might be associated with decreased nutrient intake. For patients with diabetic kidney disease, a more modest dietary protein restriction is recommended (0.6–0.8 g/kg/day) [41,42]. According to British guidelines [28] for patients with Stages 4–5 CKD not on dialysis protein intake should be as high as 0.8–1.0 g/kg ideal body weight (IBW)/day. According to these authors, there is insufficient evidence to routinely recommend low protein diets for subjects with progressive kidney disease [28]. The guidelines of highly developed countries recommend a supply of 0.6 g/kg/bw at eGFR < 50 mL/min/1.73 m^2^. The implementation of these recommendations is possible if some of the products providing vegetable protein are replaced with special low-protein products (low-protein cereal products). Therefore, these recommendations are easier to implement in countries with high levels of health care where dietary support is more readily available. In other countries, it is recommended to eat a diet with a higher protein content [27]. Despite a lot of observational studies, the lack of randomized clinical trials has prevented to recommend one type of protein over another in patients with CKD [41,42]. Nevertheless, in recent cohort study Haring et al. [58] using a food frequency questionnaire showed varied associations of specific dietary protein sources with risk of incident CKD. Red and processed meat consumption was associated with increased CKD risk while nuts, legumes and low-fat dairy products had a protective effect [58].

In our research, we assessed only total fat consumption, not by type of fatty acids. Fat intake was consistent with the Polish guidelines for patients with diabetes [56]. Similar to carbohydrates, KDOQI guidelines does not make recommendations for fat intake in patients with CKD [41,42]. The exception applies to supplementation with long chain omega-3 polyunsaturated fatty acids. There is evidence of the beneficial effects of omega-3 fatty acids on albuminuria in diabetic nephropathy [38]. Omega-3 and 6 polyunsaturated fatty acids (PUFAs) and monounsaturated fatty acids (MUFAs) were found to have beneficial impact upon DN outcomes through the attenuation of inflammation and endothelial dysfunction and improved control of hypertension and dyslipidemia [35].

In our research, we found that the consumption of potassium was within normal range, but phosphorus exceeded the recommendations both for patients with CKD stages 3–5 (800–1000 mg/d), and recommended dietary allowance for adult general population [41]. This result is close to the average consumption of phosphorus in Poland (1208 mg) [59]. In our group, serum phosphate level was within the norm. KDOQI Guidelines emphasize that serum phosphorus levels also depend on other than intestinal phosphorus absorption, so these recommendations do not suggest specific dietary phosphate ranges. They emphasize the need for individualize treatments based on patient needs and clinical judgment, taking into consideration natural sources of organic phosphorus (animal vs. vegetal protein-based dietary phosphorus) and the use of phosphorus additives in processed food [41].

Our study did not demonstrate the effect of the diet of patients on the concentration of adiponectin. Other studies found that the Mediterranean diet, the plant-based diet and diet with reduced energy value [17,18], and the DASH diet (Dietary Approaches to Stop Hypertension) [18] had a beneficial effect on adiponectin concentration. Because of the high proportion of vegetables, fruits, whole grains (i.e., complex and unrefined carbohydrates), nuts, seeds, and beans (plant proteins), low-fat dairy products and low animal proteins (especially red meat) [3,35]. These can be useful approaches in the management of hypertension, diabetes as well as CKD. Moreover, it has been shown that Mediterranean-like diet is associated with better kidney function, while low adherence may result in poorer survival rates among individuals with CKD [39]. This kind of diets are associated with lower endogenous acid (diet acids) load which may minimize individual nephron workload and slow loss of kidney function [3]. Unfortunately, these types of food are often restricted in advanced stages of CKD due to their high potassium and phosphorus content. Thus, consumption of fruits and vegetables with low potassium content, avoidance of processed convenience foods with high phosphorus content, and cooking procedures that reduce potassium and phosphorus levels in food are recommended [33,37]. It should be emphasized that only the Mediterranean and high fruit and vegetable dietary patterns had sufficient evidence to create recommendations [41,42]. The safety and acceptability of various dietary patterns must be determined on an individual basis in advanced stages of kidney disease, especially in regard to serum potassium control and adequacy of protein and energy intake [38,41,42].

## 5. Conclusions

The study confirms the high prevalence of obesity in patients with DN which may be both the cause of CKD and the cause of its complications. Excessive supply of protein was found in the patients’ diets, which may contribute to the deterioration of the course of the disease and its prognosis. In patients with eGFR > 30 there was a negative correlation between the concentration of ghrelin and WHR, creatinine and albumin, and in patients with eGFR > 30, a positive correlation between the concentration of ghrelin and vegetable protein, carbohydrates, glucose, fiber, and potassium intake. There was no relationship between adiponectin, diet, and nutritional status.

## Figures and Tables

**Table 1:** Anthropometric parameters in the study group (*n* = 55).

Anthropometric Parameter	Mean ± SD	Median	Min.	Max.
Height (cm)	164.69 ± 8.29	165.00	143.00	185.00
Body mass (kg)	83.51 ± 19.53	78.00	42.50	136.00
BMI (kg/m^2^)	30.68 ± 6.40	29.36	16.81	50.56
Waist circumference (cm)	104.20 ± 14.39	100.00	68.00	150.00
Hip circumference (cm)	108.74 ± 13.32	106.00	100.0	13.32
WHR	0.96 ± 0.07	0.95	0.81	1.11
WHtR	0.63 ± 0.08	0.61	0.43	0.99
Lean tissue mass (kg)	33.80 ± 8.97	33.3	21.30	57.90
Lean tissue mass (%)	42.54 ± 13.03	42.40	22.30	99.80
Fat mass (kg)	35.18 ± 14.20	32.40	4.20	80.00
Fat mass (%)	41.03 ± 9.08	41.50	7.20	58.80

**Table 2:** Blood parameters in the study group (*n* = 55).

Blood Parameters	Mean ± SD	Median	Min	Max
eGFR MDRD (mL/min/1.73 m^2^)	27.99 ± 10.49	26.33	8.82	70.89
Creatinine (mg/dL)	2.44 ± 0.93	2.30	1.00	6.900
Urea (mg/dL)	92.64 ± 32.04	85.50	34.04	185.00
Total protein (g/dL)	7.10 ± 0.52	7.20	6.00	8.40
Phosphorus (mg/dL)	3.44 ± 0.73	3.40	2.20	5.40
Albumin (g/dL)	4.16 ± 0.31	4.20	3.10	4.70
CRP	0.65 ± 0.86	0.300	0.100	4.900
Lymphocytes (10^9^/L)	1.72 ± 0.65	1.76	0.70	4.00
Total adiponectin (ng/mL)	2709.74 ± 2433.66	1818.34	156.27	12,832.35
Total ghrelin (pg/mL)	171.45 ± 44.44	164.01	71.77	256.95

**Table 3:** Energy and selected nutrients intake in the study group (*n* = 55).

Nutritional Parameters	Mean ± SD	Median	Min	Max
Energy (kcal)	1979.67 ± 791.84	1935.20	820.00	5032.00
Total protein (g)	70.55 ± 30.07	68.00	28.00	110.00
Vegetable protein (g)	25.31 ± 10.06	22.00	8.30	62.20
Animal protein (g)	45.24 ± 23.27	43.10	6.20	96.50
Fats (g)	63.48 ± 28,43	55.70	16.50	130.00
Carbohydrates (g)	327.09± 91.49	312.00	67.90	534.70
Glucose (g)	10.45 ± 4.60	10.70	1.10	19.30
Fructose (g)	15.11 ± 7.93	12.90	1.10	30.00
Sucrose (g)	53.62 ± 40.72	43.90	3.40	154.00
Fiber	27.79 ± 8.42	29.10	13.40	50.50
Potassium	3502.57 ± 1057.71	3566.50	1314.80	5500.00
Phosphorus	1276.84 ± 438.17	1323.00	385.60	2145.50

**Table 4:** Correlations between the selected anthropometric parameters and the concentration of ghrelin and adiponectin in two studied groups of different stage of the disease.

Anthropometric Parameters	Adiponectin			Ghrelin		
	eGFR MDRD (mL/min/1.73 m^2^)			eGFR MDRD (mL/min/1.73 m^2^)		
	<30 (N = 37)	>30 (N = 18)	Total (N = 55)	<30 (N = 37)	>30 (N = 18)	Total (N = 55)
Body mass	0.212	−0.373	−0.055	−0.207	−0.16	−0.113
BMI	0.225	−0.233	0.007	−0.074	−0.031	−0.040
Waist circumference	0.065	−0.360	−0.121	−0.296	−0.054	−0.191
Hip circumference	0.244	−0.223	0.014	−0.119	−0.089	−0.086
WHR	−0.239	−0.299	−0.260	−0.382 *	0.039	−0.238
WHtR	0.056	−0.210	−0.065	−0.181	−0.072	−0.128
Lean tissue mass (kg)	−0.191	−0.376	−0.259	−0.342	0.111	−0.206
Lean tissue mass (%)	−0.170	0.154	−0.077	−0.047	0.124	−0.016
Fat mass (kg)	0.276	−0.319	0.070	−0.100	−0.082	−0.074
Fat mass (%)	0.161	−0.216	0.040	−0.015	−0.156	−0.040

* Correlation is significant at the 0.05 level (2-tailed).

**Table 5:** Correlations between the energy and selected nutrients intake and the concentration of ghrelin and adiponectin in two studied groups of different stage of the disease.

Energy and Nutrients Intake	Adiponectin			Ghrelin		
	eGFR MDRD (ml/min/1.73 m^2^)			eGFR MDRD (ml/min/1.73 m^2^)		
	<30 (N = 37)	>30 (N = 18)	Total (N = 55)	<30 (N = 37)	>30 (N = 18)	Total (N = 55)
Energy	0.141	−0.191	−0.105	0.137	0.078	0.088
Protein	0.202	−0.348	−0.020	0.073	0.467	0.204
Vegetable protein	0.001	−0.271	−0.067	0.115	0.610 **	0.225
Animal protein	0.238	−0.394	−0.013	0.021	0.322	0.122
Carbohydrates	0.109	−0.283	−0.043	0.184	0.565 *	0.304 *
Glucose	−0.017	−0.203	−0.084	0.052	0.521 *	0.196
Fructose	−0.046	−0.280	−0.123	0.021	0.329	0.112
Sucrose	0.245	−0.202	0.028	0.256	0.421	0.321 *
Fats	0.169	−0.365	−0.052	0.114	0.329	0.188
Fiber	−0.131	−0.434	−0.226	−0.015	0.498 *	0.138
Potassium	0.110	−0.285	−0.039	−0.015	0.536 *	0.164
Phosphorus	0.092	−0.398	−0.085	−0.021	0.355	0.090

* Correlation is significant at the 0.05 level (2-tailed), ** correlation is significant at the 0.01 level (2-tailed).

## Data Availability

The data presented in this study are available upon request from the corresponding author. The data are not publicly available as they are still being used for analysis and manuscript writing.

## References

[1] GBD Chronic Kidney Disease Collaboration (2020). Global, regional, and national burden of chronic kidney disease, 1990–2017: A systematic analysis for the Global Burden of Disease Study 2017. Lancet.

[2] Gubina N.V., Kupnovytska I.H., Mishchuk V.H., Markiv H.D. (2020). Ghrelin levels and decreased kidney function in patients with early stages of chronic kidney disease against the background of obesity. J. Med. Life.

[3] Kramer H. (2019). Diet and chronic kidney disease. Adv. Nutr..

[4] DiNicolantonio J.J., Bhutani J., O’Keefe J.H. (2016). Added sugars drive chronic kidney disease and its consequences: A comprehensive review. J. Insul. Resist..

[5] Dornas W.C., de Lima W.G., Pedrosa M.L., Silva M.E. (2015). Health implications of high-fructose intake and current research. Adv. Nutr..

[6] Vieira F.O., de Oliveira Leal V., Barcza Stockler-Pinto M., de Faria Barros A., Borges N.A., Lobo J.C., Mafra D. (2015). Fructose intake: Is there an association with uric acid levels in nondialysis-dependent chronic kidney disease patients?. Nutr. Hosp..

[7] Arslan S., Sanlier N. (2016). Relationship between daily dietary fructose intake, body composition and biochemical parameters patients with type 2 diabetes. J. Hum. Sci..

[8] Kretowicz M., Johnson R.J., Ishimoto T., Nakagawa T., Manitius J. (2011). The impact of fructose on renal function and blood pressure. Int. J. Nephrol..

[9] Donderski R., Miśkowiec-Wiśniewska I., Kretowicz M., Grajewska M., Manitius J., Kamińska A., Junik R., Joanna Siódmiak J., Stefańska A., Odrowąż-Sypniewska G. (2015). The fructose tolerance test in patients with chronic kidney disease and metabolic syndrome in comparison to healthy control. BMC Nephrol..

[10] Livesey G., Taylor R. (2008). Fructose consumption and consequences for glycation, plasma triacylglycerol, and body weight: Meta-analyses and meta-regression models of intervention studies. Am. J. Clin. Nutr..

[11] Stanhope K.L., Peter J., Havel P.J. (2010). Fructose consumption: Recent results and their potential implications. Ann. N. Y. Acad. Sci..

[12] Przybyciński J., Dziedziejko V., Puchałowicz K., Domański L., Pawlik A. (2020). Adiponectin in chronic kidney disease. Int. J. Mol. Sci..

[13] Heidari M., Nasri P., Nasri H. (2015). Adiponectin and chronic kidney disease; a review on recent findings. J. Nephropharmacol..

[14] Christou G.A., Kiortsis D.N. (2014). The role of adiponectin in renal physiology and development of albuminuria. J. Endocrinol..

[15] Perri A., Vizza D., Lofaro D., Gigliotti P., Leone F., Brunelli E., Malivindi R., De Amicis F., Romeo F., De Stefano R. (2013). Adiponectin is expressed and secreted by renal tubular epithelial cells. J. Nephrol..

[16] Kahlmann V., Manansala M., Moor C.C., Shahrara S., Wijsenbeek M.S., Sweiss N.J. (2021). COVID-19 infection in patients with sarcoidosis: Susceptibility and clinical outcomes. Curr. Opin. Pulm. Med..

[17] Izadi V., Azadbakht L. (2015). Specific dietary patterns and concentrations of adiponectin. J. Res. Med. Sci..

[18] Janiszewska J., Ostrowska J., Szostak-Wegierek D. (2021). The influence of nutrition on adiponectin—A narrative review. Nutrients.

[19] Aydemir N., Pike M.M., Alsouqi A., Headley S.A.E., Tuttle K., Evans E.E., Milch C.M., Moody K.A., Germain M., Lipworth L. (2020). Effects of diet and exercise on adipocytokine levels in patients with moderate to severe chronic kidney disease. Nutr. Metab. Cardiovasc. Dis..

[20] Kim H.Y., Bae E.H., Ma S.K., Chae D.W., Choi K.H., Kim Y.S., Hwang Y.H., Ahn C., Kim S.W. (2016). Association of serum adiponectin level with albuminuria in chronic kidney disease patients. Clin. Exp. Nephrol..

[21] Song S.H., Oh T.R., Choi H.S., Kim C.S., Ma S.K., Oh K.H., Ahn C., Kim S.W., Bae E.H. (2020). High serum adiponectin as a biomarker of renal dysfunction: Results from the KNOW-CKD study. Sci Rep..

[22] Yoshimoto A., Mori K., Sugawara A., Mukoyama M., Yahata K., Suganami T., Takaya K., Hosoda H., Kojima M., Kangawa K. (2002). Plasma ghrelin and desacyl ghrelin concentrations in renal failure. J. Am. Soc. Nephrol..

[23] El-Khashab S.O., Behiry M.E. (2019). Adiponectin and ghrelin: Nutritional regulatory role in chronic kidney disease patients. Egypt. J. Intern. Med..

[24] Boniecka I., Jeznach -Steinhagen A., Szostak-Węgierek D., Rymarz A., Niemczyk S. (2019). Ghrelin and its role in chronic kidney disease. Przegląd Lekarski..

[25] Lv Y., Liang T., Wang G., Li Z. (2018). Ghrelin, a gastrointestinal hormone, regulates energy balance and lipid metabolism. Biosci. Rep..

[26] Suzuki H., Asakawa A., Amitani H., Nakamura N., Inui A. (2013). Ghrelin and cachexia in chronic kidney disease. Pediatr. Nephrol..

[27] Małgorzewicz S., Ciechanowski K., Kozłowska L., Krzanowska K., Krzanowski M., Kaczkan M., Borek P., Jankowska M., Rutkowski B., Dębska-ślizień A. (2019). Zasady żywienia w przewlekłej chorobie nerek—Stanowisko Grupy Roboczej Polskiego Towarzystwa Nefrologicznego. Forum Nefrol..

[28] Wright M., Southcott E., MacLaughlin H., Wineberg S. (2019). Clinical practice guideline on undernutrition in chronic kidney disease. BMC Nephrol..

[29] Anupama S.H., Abraham G., Alex M., Vijayan M., Subramanian K.K., Fernando E., Nagarajan V., Nageshwara Rao P.K. (2020). A multicenter study of malnutrition status in chronic kidney disease stages I-V-D from different socioeconomic groups. Saudi J. Kidney Dis. Transpl..

[30] Apetrii M., Timofte D., Voroneanu L., Covic A. (2021). nutrition in chronic kidney disease-The role of proteins and specific diets. Nutrients.

[31] Gebretsadik G.G., Mengistu Z.D., Molla B.W., Desta H.T. (2020). Patients with chronic kidney disease are not well adhered to dietary recommendations: A cross-sectional study. BMC Nutr..

[32] Iorember F.M. (2018). Malnutrition in chronic kidney disease. Front. Pediatr..

[33] Kalantar-Zadeh K., Fouque D. (2017). Nutritional management of chronic kidney disease. N. Engl. J. Med..

[34] Akchurin O.M. (2019). Malnutrition in chronic kidney disease. Pediatr. Clin. N. Am..

[35] Ko G.J., Kalantar-Zadeh K., Goldstein-Fuchs J., Rhee C.M. (2017). Dietary approaches in the management of diabetic patients with kidney disease. Nutrients.

[36] KDOQI (2007). KDOQI clinical practice guidelines and clinical practice recommendations for diabetes and chronic kidney disease. Am. J. Kidney.

[37] Anderson C.A.M., Nguyen H.A. (2018). Nutrition education in the care of patients with chronic kidney disease and end-stage renal disease. Semin. Dial..

[38] Goldstein-Fuchs J., Kalantar-Zadeh K. (2015). Nutrition intervention for advanced stages of diabetic kidney disease. Diabetes Spectr..

[39] Rysz J., Franczyk B., Ciałkowska-Rysz A., Gluba-Brzózka A. (2017). The effect of diet on the survival of patients with chronic kidney disease. Nutrients.

[40] Therrien M., Byham-Gray L., Beto J. (2015). A review of dietary intake studies in maintenance dialysis patients. J. Ren. Nutr..

[41] Ikizler T.A., Burrowes J.D., Byham-Gray L.D., Campbell K.L., Carrero J.J., Chan W., Fouque D., Friedman A.N., Ghaddar S., Goldstein-Fuchs D.J. (2020). KDOQI clinical practice guideline for nutrition in CKD: 2020 update. Am. J. Kidney Dis..

[42] Ikizler T.A., Cuppari L. (2021). The 2020 updated KDOQI clinical practice guidelines for nutrition in chronic kidney disease. Blood Purif..

[43] Włodarek D., Głąbska D., Rojek-Trębicka J. (2014). Assessment of diet in chronic kidney disease female predialysis patients. Ann. Agric. Environ. Med..

[44] Levey A., Bosch J., Lewis J. (1999). A more accurate method to estimate glomerular filtration rate from serum creatinine: A new prediction equation. Modification of diet in renal disease study group. Ann. Intern. Med..

[45] Król E., Rutkowski B. (2008). Przewlekła choroba nerek—Klasyfikacja, epidemiologia i diagnostyka. Forum. Nefrol..

[46] World Health Organization (WHO) (1997). Report of a WHO Consultation on Obesity. Obesity: Preventing and Managing the Global Epidemic.

[47] WHO (2011). Waist Circumference and Waist–Hip Ratio: REPORT of a WHO Expert Consultation, Geneva, 8–11 December 2008.

[48] Alberti K.G., Eckel R.H., Grundy S.M., Zimmet P.Z., Cleeman J.I., Donato K.A., Fruchart J.C., James W.P., Loria C.M., Smith S.C. (2009). Harmonizing the Metabolic Syndrome A Joint Interim Statement of the International Diabetes Federation Task Force on Epidemiology and Prevention; Hational Heart, Lung, and Blood Institute; American Heart Association; World Heart Federation; International Atherosclerosis Society; International Association for the Study of Obesity. Circulation.

[49] Ashwell M., Gunn P., Gibson S. (2012). Waist-to-height ratio is a better screening tool than waist circumference and BMI for adult cardiometabolic risk factors: Systematic review and meta-analysis. Obes. Rev..

[50] Ashwell M., Gibson S. (2016). Waist-to-height ratio as an indicator of ‘early health risk’: Simpler and more predictive than using a ‘matrix’ based on BMI and waist circumference. BMJ Open.

[51] Jeznach-Steinhagen A., Czerwonogrodzka-Senczyna A., Jeznach-Steinhagen A. (2020). Wybrane metody oceny stanu odżywienia i sposobu żywienia pacjenta z cukrzycą. Żywienie osób z Cukrzyca i Chorobami Towarzyszącymi.

[52] Canpolat N., Sever L., Agbas A., Tasdemir M., Oruc C., Ekmekci O.B., Caliskan S. (2018). Leptin and ghrelin in chronic kidney disease: Their associations with protein-energy wasting. Pediatric. Nephrol..

[53] Sahathevan S., Khor B.H., Ng H.M., Gafor A.H.A., Mat Daud Z.A., Mafra D., Karupaiah T. (2020). Understanding development of malnutrition in hemodialysis patients: A narrative review. Nutrients.

[54] Sekar M., Elumala R., Kempanahalli B., Manech K., Lakkakula Bhaskar V.K.S., Periasamy S. (2015). Effect of chronic kidney disease on circulation grelin concentrations. Ann. Rom. Soc. Cell Biol..

[55] World Health Organization (2015). Guideline: Sugars Intake for Adults and Children.

[56] Araszkiewicz A., Bandurska-Stankiewicz E., Borys S., Budzyński A., Cyganek K., Cypryk K., Czech A., Czupryniak L., Drzewoski J., Dzida G. (2021). 2021 Guidelines on the management of patients with diabetes. A position of Diabetes Poland. Clin. Diabetol..

[57] Livesey G., Taylor R., Livesey H.F., Buyken A.E., Jenkins D.J.A., Augustin L.S.A., Sievenpiper J.L., Barclay A.W., Liu S., Wolever T.M.S. (2019). Dietary glycemic index and load and the risk of type 2 diabetes: A systematic review and updated meta-analyses of prospective cohort studies. Nutrients.

[58] Haring B., Selvin E., Liang M., Coresh J., Grams M.E., Petruski-Ivleva N., Steffen L.M., Rebholz C.M. (2017). Dietary protein sources and risk for incident chronic kidney disease: Results from the Atherosclerosis Risk in Communities (ARIC) study. J. Ren. Nutr..

[59] Wojtasik A., Wożniak A., Stoś K., Jarosz M., Rychlik E., Stoś K., Charzewska J. (2020). Składniki mineralne. Normy Żywienia Dla Populacji Polski I Ich Zastosowanie.

